# UBQLN2 in neurodegenerative disease: mechanistic insights and emerging therapeutic potential

**DOI:** 10.1042/BST20253053

**Published:** 2025-07-14

**Authors:** Autumn M. Matthews, Alexandra M. Whiteley

**Affiliations:** Department of Biochemistry, University of Colorado Boulder, Boulder, Colorado, 80309, U.S.A.

**Keywords:** ALS, mitochondria, neurodegenerative disease, PEG10, protein degradation, stress granules, Ubiquilin 2

## Abstract

Ubiquilins (UBQLNs) regulate cellular protein turnover by shuttling proteins, or ‘clients’, to the proteasome or autophagy pathways for degradation. Of the five different *UBQLN* genes in humans, *UBQLN2* is the most highly expressed in the nervous system and muscle tissue and has been linked to multiple neurodegenerative diseases. In particular, point mutations of *UBQLN2* cause an X-linked, dominant form of amyotrophic lateral sclerosis (ALS), ALS with frontotemporal dementia (ALS/FTD), or FTD. Failed protein degradation is a hallmark of many neurodegenerative diseases, including ALS and FTD; however, it is not clear exactly how ALS/FTD-associated *UBQLN2* mutations contribute to pathogenesis. Recent studies have revealed the complexity of UBQLN2 biology and allow deeper understanding as to how UBQLN2 dysfunction may contribute to neurodegenerative disease. UBQLN2 is necessary for mitochondrial protein degradation and for regulating mitochondrial turnover, both of which are essential for motor neurons and have been implicated in the pathogenesis of ALS. Stress granule (SG) formation and regulation are also affected by UBQLN2 mutations, and their dysregulation may contribute to the toxic protein aggregation and SG changes observed in neurodegenerative disease. Finally, there are compelling links connecting UBQLN2 dysfunction with changes to downstream neuronal morphology, function, and behavior. This review will detail the emerging consensus on how UBQLN2 protects against neurodegenerative disease and will provide insights into potential therapeutic approaches.

## Introduction

The degradation of proteins is essential for maintaining cellular health, and impaired removal can lead to protein aggregation, dysfunction of the cell, and proteotoxicity [1]. Some cells are uniquely sensitive to perturbation in protein degradation systems, which can lead to disease when degradation pathways fail. For example, failed protein degradation is a commonly observed hallmark of many neurodegenerative diseases, and in some cases, mutation of genes involved in protein degradation is their ultimate etiology [2–6].

**Figure 1:**
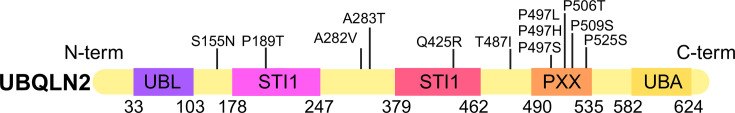
Schematic of UBQLN2 protein. Simplified schematic showing UBQLN2 domains, with a variety of disease-causing mutations annotated. The beginning and end of each protein domain is shown at the bottom with amino acid residues. PXX, proline-rich repeat domain; STI1, stress-induced protein 1-like motif; UBA, ubiquitin-associated domain; UBL, ubiquitin-like domain.

Ubiquilins (UBQLNs) are a family of proteasomal shuttle factors that are thought to regulate the degradation of protein ‘clients’ via the ubiquitin-proteasome system (UPS) and autophagy [7–11], and particular focus has been applied to their role in ER-associated degradation [12,13]. There are five *UBQLN* genes in humans that share a high level of sequence homology but differ largely in their tissue expression patterns: *UBQLN1* and *UBQLN4* are ubiquitously expressed, *UBQLN2* is highly expressed in neural tissue and muscle, and *UBQLN3* and *UBQLNL* are testis-specific [8,12,14]. UBQLNs share conserved protein domains, including an N-terminal ubiquitin-like domain (UBL) that binds to the regulatory cap of the proteasome [9], one or two stress-induced protein 1 (STI1) domains that mediate protein–protein interactions [15,16], and a ubiquitin-associated (UBA) domain that binds to ubiquitin chains with little preference for length or chain linkage type [8,17–19] (Figure 1). UBQLN2 uniquely contains a proline-rich repeat (PXX) domain just before the UBA domain. This short domain is approximately 50 amino acids in length and is believed to contribute to UBQLN2’s ability to promote phase separation, as well as binding to client proteins and the proteasome [20,21]. While the circumstances that render UBQLN function necessary in different proteostasis pathways remain poorly understood, the current model of UBQLN function is that their simultaneous binding of client, proteasome cap, and/or LC3 promotes delivery of those clients to degradation machinery [7,21,22].

The selectivity of UBQLNs for particular client proteins also remains murky, with evidence supporting UBQLN-mediated degradation of aggregated [23], hydrophobic [15,24], mislocalized [25–27], and neurodegeneration-associated proteins including α-synuclein, presenilins, huntingtin, TDP-43, and others [8,28–33]. Furthermore, although the role of UBQLN2 on facilitating protein degradation is well documented [8,15,23–33], recent work has shown a role for UBQLN2 in protecting some client proteins from degradation [34,35]. The ability of UBQLN2 to control protein fate by shuttling clients toward degradative or nondegradative outcomes further complicates our still growing understanding of UBQLN2 biology.

Multiple *UBQLN* genes have been associated with neurodevelopmental [36] and neurodegenerative diseases, including synucleinopathies [28,37], Alzheimer’s Disease [38], and amyotrophic lateral sclerosis (ALS) [39,40] through either etiological mutation or associated changes to gene expression. The strongest connection of UBQLNs to neurodegenerative disease is between UBQLN2 and ALS with frontotemporal dementia (FTD) [39,40]. ALS is a rare, fatal neurodegenerative disease characterized by degeneration of motor neurons in the brain and spinal cord, which is sometimes accompanied by degeneration of the frontal cortex (ALS/FTD) [41]. In total, 90% of ALS cases are sporadic, whereas 10% are familial (referred to as fALS) and are caused by mutation of genes including *C9orf72, SOD1*, and many others [39,41,42]. While the specific molecular defects downstream of UBQLN2 mutation that lead to ALS, ALS/FTD, or FTD remain unclear, a growing understanding of UBQLN2 function has provided clues as to the molecular etiology of disease. This review will highlight the recent advances in our understanding of UBQLN2 biology, how disease-linked mutations may be pathogenic, and the therapeutic potential targeting these dysregulated pathways.

### Wildtype and mutant UBQLN2 are associated with neurodegenerative disease

In the first study to identify *UBQLN2* mutations in fALS, five *UBQLN2* point mutations were found in tight association with the disease, all falling within the unique PXX domain (P497S, P497H, P506T, P509S, and P525S) of UBQLN2 [39]. Since this discovery, many other disease-associated *UBQLN2* mutations have been identified, with some of these mutations falling outside the PXX domain [4,40,43,44] (Figure 1). While most of these mutations are associated with ALS or ALS/FTD, one identified mutation, A282V, is associated with a pure FTD phenotype, although it remains unknown whether this particular disease presentation is due to the location of this mutation in the *UBQLN2* gene or other factors [40].

*UBQLN2* is on the X chromosome, and given the X-linked, dominant pattern of heritability of *UBQLN2*-mediated ALS/FTD [39], it is thought that mutation of UBQLN2 results in a toxic gain-of-function phenotype. In agreement with these findings, UBQLN2-positive aggregates are observed in histological specimens of *UBQLN2*-mediated fALS [45]. In addition, mutant forms of UBQLN2 aggregate and phase-separate in cells and *in vitro* [46–48]*,* suggesting that an aspect of the gain-of-function involves predisposition to aggregation. However, even in the absence of mutation, UBQLN2-positive aggregates have been observed in sporadic ALS and in fALS associated with *SOD1, TDP-43, and C9orf72* mutations [39,45,49]. Intense UBQLN2 staining has also been observed in Lewy bodies, the defining pathological characteristic of Parkinson’s disease and dementia with Lewy bodies [37,50]. Together, these suggest a more widespread phenomenon of UBQLN2 co-aggregation in neurodegenerative disease.

Cellular evidence suggests that mutation of *UBQLN2* results in a toxic gain-of-function, as well as a loss-of-function, phenotype through the seeding of protein aggregates paired with an inability to facilitate protein degradation [23,27,48,51–54]. In cells, disease-linked mutations of *UBQLN2* mimic UBQLN2 depletion by causing proteotoxic stress and protein accumulation [23]. It should be noted, however, that *Ubqln2* ^−/−^ mice exhibit a comparatively mild form of neurodegenerative disease, suggesting that the gain-of-function due to mutation contributes significantly to the disease phenotype observed [29,55–57].

### UBQLN2 disruption causes widespread changes to protein degradation pathways

At its most basic level, it has been thought that the major defect upon *UBQLN2* mutation is perturbation to protein degradation pathways, and that neuronal dysfunction lays somewhere downstream of proteome dysregulation. In support of this model, expression of mutant *UBQLN2* alleles in cell culture systems leads to a rapid accumulation of polyubiquitinated proteins [21,58]. The reason for protein accumulation appears to be multifactorial: for example, cell culture experiments have shown a predisposition of mutant UBQLN2 to aggregate [48,56,59] and a decreased ability to bind to the proteasome [21], as well as cofactors such as Hsp70 [23]. In addition, mutant UBQLN2 exhibits altered phase separation dynamics [20,46,47,55,60]. However, it remains unclear which of these effects seen in particular cell culture conditions and cell lines are broadly shared with neurons in culture or *in vivo*.

Animal models of *UBQLN2*-mediated ALS also show large-scale changes to protein degradation pathways [23,29,53,55,57]. Mutant knock-in mice, which express endogenous levels of a humanized mutant allele, accumulate protein aggregates and demonstrate cognitive deficits [23]. *UBQLN2* transgenic (Tg) mouse lines, which overexpress either a WT or disease-associated human *UBQLN2* allele, exhibit more dramatic phenotypes involving both behavior and neuromotor function, eventually resulting in hind limb paralysis [29]. These mice showed changes to the abundance of p62, suggesting either failed degradation or upregulation of compensatory degradation pathways [53,57]. Mutant UBQLN2 and p62, colocalized by histological analysis [53], have also been observed in patient brain tissues of *UBQLN2*-linked fALS [45]. Overexpression of mutant UBQLN2 also caused a decrease in the vacuolar ATPase ATP6v1g1, further linking UBQLN2 with autophagosome acidification, and ultimately, autophagy [53]. In comprehensive analyses of Tg, knock-in, and knockout *UBQLN2* mouse models, all models demonstrated altered protein degradation dynamics, with particular perturbations to proteasomal proteins, E3 ligases, and p62 [57]. In addition to mouse models, expression of UBQLN2^P497H^ in the spinal cords of rats caused key features of ALS, including progressive degeneration of motor neurons and protein accumulation, further linking dysregulation to proteostasis pathways [54].

While it is clear that mutant UBQLN2 disrupts proteostasis, the particular client proteins most influenced by this disruption have remained poorly understood. Unbiased global proteomics of hippocampal and lumbar spinal cord samples from *Ubqln2^−/−^*, WT UBQLN2, *UBQLN2* ^P506T^ knock-in, or *UBQLN2* ^P497S^ Tg mice all show perturbation to mitochondrial protein levels, implying that mitochondrial proteins may be uniquely affected by UBQLN2 dysfunction [57]. The same proteomic study revealed the dysregulation of the proteins PEG10 and RTL8 [57], both of which are ancient, retrotransposon-derived genes [61–63] with poorly understood function [64–66] that strongly colocalize with stress granules (SGs) [67]. More detailed biochemical approaches demonstrated that UBQLN2 selectively regulates the degradation of a longer, enzyme-containing form of PEG10, through the help of RTL8 binding to UBQLN2, which may contribute to neuronal health [68,69].

Previous work also shows that UBQLN2 is capable of regulating proteins known to contribute to neurodegenerative diseases. Knockout of *UBQLN2* dysregulates α-synuclein [28]. UBQLN2 binds to TDP-43, and overexpression of UBQLN2 in cell culture systems reduces the amount of both WT and pathological TDP-43 protein [29,52,70,71]. UBQLN2 also regulates tau levels, in a mechanism dependent on the PXX domain [72]. It should be noted, however, that the dysregulation of these particular proteins is not the only – and perhaps not even the dominant – function of UBQLN2, as global analyses of multiple model systems fail to demonstrate large changes to these proteins in cells or tissues with dysfunctional UBQLN2 [60].

**Figure 2:**
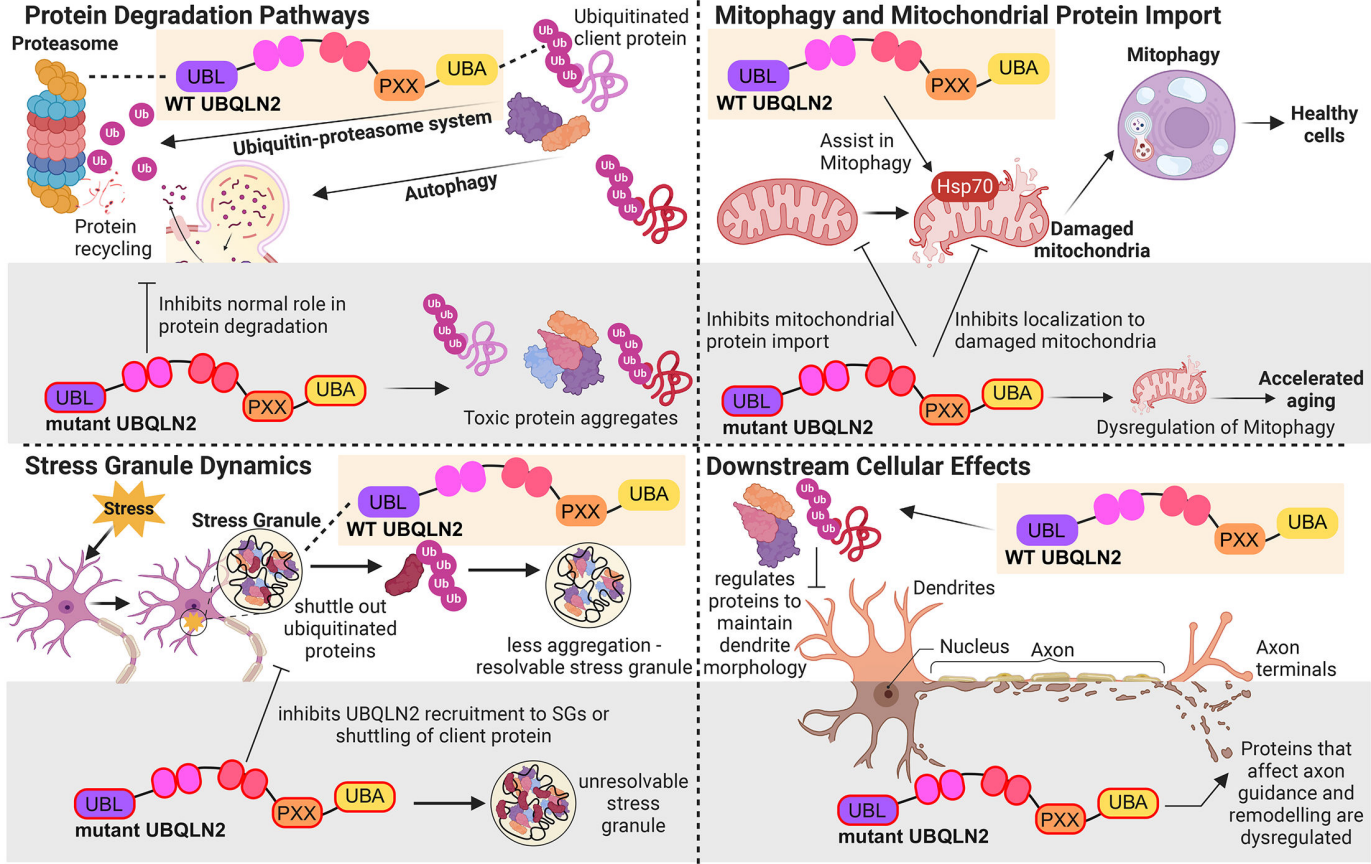
Overview of UBQLN2 dysfunction. Simplified schematic of the role of UBQLN2, and the consequences of neurodegenerative disease-associated UBQLN2 mutations, on protein degradation pathways, mitophagy and mitochondrial protein import, stress granule dynamics, and other downstream cellular effects. PXX, proline-rich repeat; STI1, stress-induced protein 1-like motif; UBA, ubiquitin-associated domain; UBL, ubiquitin-like domain.

In any case, the missing link is how failed protein degradation either at the single client level or at the global level of proteome destabilization results in the dysfunction and death of motor and cortical neurons. In Figure 2, we outline possible connections that reveal promising avenues for further investigation: (1) altered protein degradation, (2) mitochondrial failure, (3) SG regulation and dynamics, and (4) downstream effects on cellular behavior and neuronal function.

### UBQLN2 mutations lead to disruption of mitochondrial protein degradation and dynamics

Mitochondrial dysfunction is an early observation in many ALS model systems, and motor neurons of ALS patients show abnormal mitochondrial morphology, turnover, and respiration [73]. Data from animal models and cell culture systems point to a role for UBQLN2 in the maintenance of healthy mitochondria, linking UBQLN2 mutation to a known pathway in ALS.

UBQLN1 and UBQLN2 have been shown to facilitate the proteasomal degradation of mislocalized mitochondrial proteins and in some circumstances, to protect the same proteins from degradation [25,26,34]. *UBQLN2^−/−^* HeLa cells and *UBQLN2* ^P497S^ mice show a decrease in TIMM44, which is necessary for mitochondrial protein import and may potentiate problems of mislocalized protein degradation by causing their accumulation in the cytosol [27]. UBQLN2 has been further implicated in mitophagy. UBQLN2 and HSP70 colocalize at damaged mitochondria; however, UBQLN2^P497H^ and UBQLN2^P509S^ fail to colocalize, and the clearance of damaged mitochondria is impaired [74]. Perhaps as a result of impaired clearance, mitochondria of motor neurons in *UBQLN2* mutant mice, as well as *UBQLN2^−/−^* HeLa cells, are shorter, have distorted cristae with large voids, and show defects in mitochondrial respiration [27,75]. As motor neurons are uniquely susceptible to mitochondrial stress [76], *UBQLN2* mutations may be especially toxic to this cell type, thereby contributing to the specific neuronal loss and symptoms observed in ALS/FTD [76,77].

### UBQLN2 disruption leads to downstream effects on stress granule regulation and dynamics

Biomolecular condensates are densely packed cellular compartments that act as hubs for biological processes including transcription, translation, RNA metabolism, DNA damage response, and protein folding [48,78]. While there is growing evidence that disruption of condensates may generally contribute mechanistically to neurodegenerative disease [35,47,56,78,79], SGs are of particular interest as they have been widely implicated in neurodegenerative diseases, such as ALS [45,80–82].

SGs are dynamic, membraneless organelles that form in response to stress to sequester mRNA, RNA-binding proteins, translation initiation factors, and small ribosomal subunits [83,84]. Although they can be a normal response to cellular stress, SGs observed in neurodegenerative conditions are thought to contribute to disease when their persistence and inability to resolve leads to aggregation of RNA-binding proteins, which ultimately disrupts cellular function [85]. Particularly, mutations in RNA-binding proteins that mediate SG formation have been linked to ALS and/or FTD [86]. Among human UBQLN proteins, UBQLN2 localizes to SGs induced by a variety of stressors [20,47,80,87] in a mechanism that appears to be dependent on the retrotransposon-derived genes RTL8 and PEG10 [67]. UBQLN2^P497H^ negatively regulates SG formation [80,82], and similar work has shown that UBQLN2^A282V^, UBQLN2^M446R^, UBQLN2^P497H^, and especially UBQLN2^P506T^ mutations led to a significant decrease in stress-induced UBQLN2 cytoplasmic, round puncta per cell [47]. Of the stress-induced puncta that remain, fluorescence recovery after photobleaching experiments [88] reveal that UBQLN2^A282V^, UBQLN2^P497H^, and UBQLN2^P497S^ led to decreased levels of UBQLN2 mobility and less recovery than WT UBQLN2 [47,48]. While this work represents changes to stress-induced UBQLN2 puncta, the effects of these mutations may similarly influence SG formation and dynamics.

UBQLN2 is also thought to aid in the remodeling and resolution of SGs by shuttling SG proteins to degradation machinery [20], much like the ALS-linked regulator of degradation VCP [89]. In this model, ALS-causing *UBQLN2* mutations may prevent the clearance of SGs, leading to toxic protein aggregation, impaired RNA metabolism, and persistent SGs. Further research is required to confirm these findings and evaluate downstream effects of UBQLN2 on SG formation and dynamics and how this relates to neurodegenerative disease.

### Downstream effects on cell behavior and neuronal function

Degeneration of dendritic spines and axonal regression are found in ALS patient tissues [90] and in the popular SOD1^G93A^ mouse model of disease [91]. This degeneration is linked to glutamate-induced excitotoxicity [91,92], which may contribute to selective death of motor neurons as they are especially vulnerable to dysregulation of glutamate levels [92,93]. Additionally, dendritic spinopathy leads to changes to neuronal signaling that are believed to contribute to ALS phenotypes [94].

Patient tissue and animal models of *UBQLN2*-mediated ALS/FTD also demonstrate dendritic spinopathy [39,94], and *Ubqln2* ^−/−^ mice exhibit decreased serotonin receptor positivity in spinal cord and brain sections, further linking changes in protein degradation with neurotransmitter abundance [57]. In cell culture, differentiated motor neurons expressing a mutant *UBQLN2* allele with four ALS-associated mutations (*UBQLN2^4XALS^*) showed a significant reduction in total neurite length and complexity [95]. Additionally*, Drosophila* expressing *UBQLN2^4XALS^* showed a rough eye phenotype that is suggestive of eye degeneration [95]. Silencing of *Unc-5,* a gene that plays a key role in mediating axonal repulsion, suppressed UBQLN2-associated eye degeneration and increased UBQLN2^4XALS^ fly lifespan [95], suggesting an important role of UBQLN2 on regulating proteins in axon extension pathways.

While the precise link between failed protein degradation and dendritic spinopathy remains unclear, a possible connection is the relationship between UBQLN2 and the virus-like protein PEG10 [68]. UBQLN2 regulates the proteasomal degradation of a form of PEG10 that contains a protease domain necessary for PEG10 self-cleavage. Self-cleavage of PEG10 generates a short protein fragment which contains an RNA-binding domain which localizes to the nucleus and alters transcript levels of genes in neuronal pathways, including axon extension, axon guidance, and neuronal projection [68]. The inability of mutant UBQLN2 to regulate PEG10, therefore, provides a compelling link to the derangement of neuronal function and the symptoms of ALS/FTD, although future work is needed to determine the exact contribution of PEG10 to neuronal dysfunction.

### Therapeutic implications of UBQLN2 in neurodegeneration

**Figure 3:**
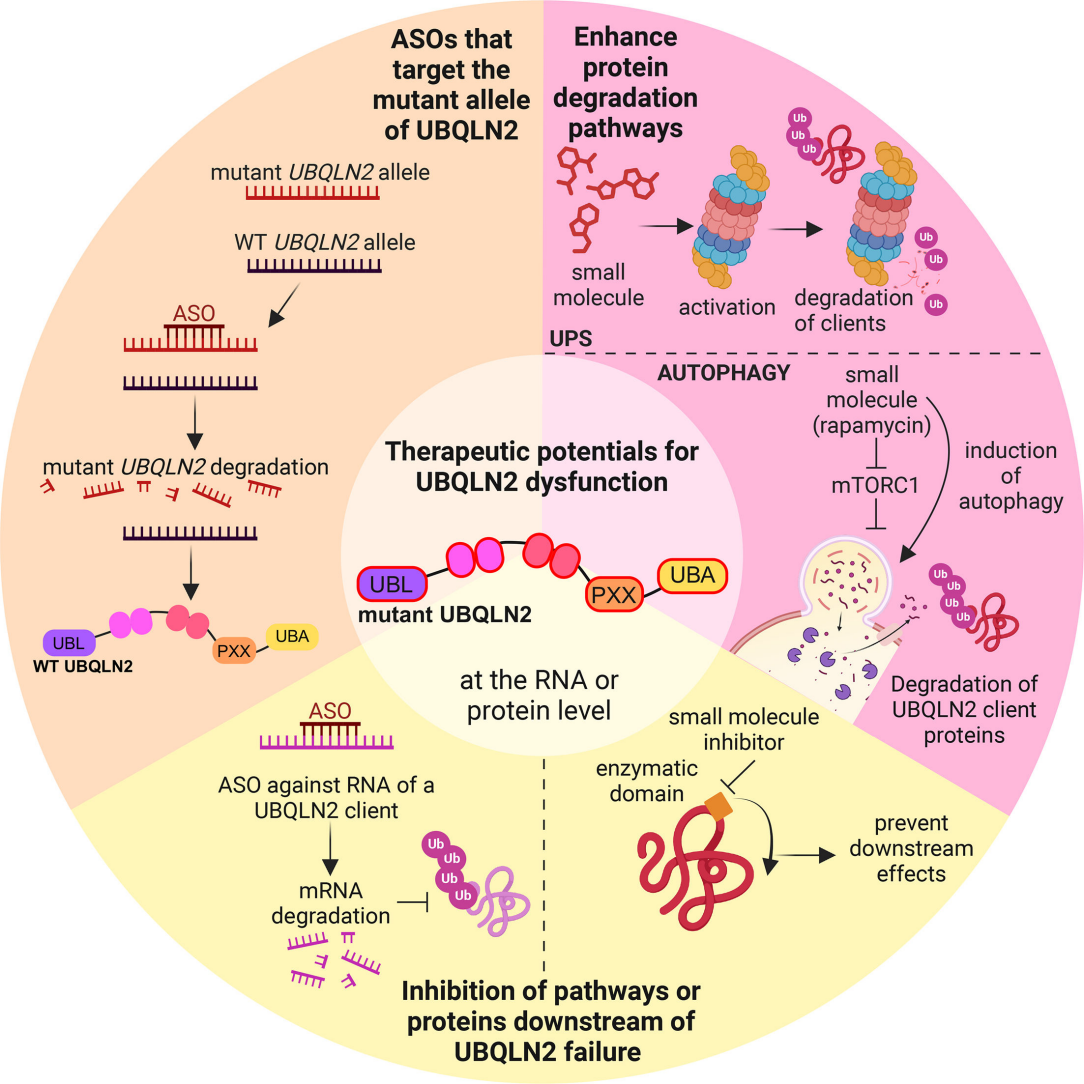
Proposed therapeutic targets for *UBQLN2*-mediated neurodegenerative disease. Simplified schematic of potential therapeutics that target the mutant allele of *UBQLN2*, enhance protein degradation capacity, or inhibit downstream effects of UBQLN2 dysfunction. ASOs, antisense oligonucleotides; PXX, proline-rich repeat; UBA, ubiquitin-associated domain; UBL, ubiquitin-like domain.

Given the role of UBQLN2 in multiple pathways linked to neurodegeneration, targeting of UBQLN2 and its downstream effects are attractive for therapeutic development. UBQLN2-targeted therapies fall into three major categories: (1) antisense oligonucleotides (ASOs) that target mutant alleles of *UBQLN2*, (2) therapies that enhance protein degradation pathways and reduce the requirement for UBQLN2-mediated degradation, and (3) inhibition of pathways or proteins downstream of UBQLN2 failure (Figure 3).

ASOs are short oligonucleotides that bind to RNA and can modify gene expression either through altered splicing, translation, or targeted degradation of transcript [96,97]. ASOs can be generated against specific mutant alleles of *UBQLN2,* which would result in a decrease in the expression of the mutant allele, thereby preventing the toxic gain-of-function effects. A limitation of this approach is that because *UBQLN2* ^−/−^ models also demonstrate protein degradation defects, maintenance of WT *UBQLN2* allele expression may be important. In particular, because *UBQLN2* is on the X chromosome, an ASO-based therapeutic approach could differentially affect males versus females.

To combat dysregulation of proteostasis pathways, induction of UPS and autophagy function could also be a strategy for therapeutic intervention [1] by minimizing the requirements for UBQLN2 function. This remains an active area of development, but there are already multiple promising small molecules, some of which are already FDA-approved therapeutics, that modulate proteasome activity [98,99]. As an alternative, autophagy can also be activated to degrade misfolded or aggregated proteins, using small molecules [100–102].

Therapeutic approaches can also be targeted to clients of UBQLN2 that are thought to play a direct role in the progression of disease. For example, ASOs could be used to decrease a client protein’s abundance when UBQLN2 is unable to control its degradation. Similarly, knockdown of client proteins using targeted degraders could be effective and is in the development for ALS-related proteins including TDP-43 [2]. Finally, clients with unique functions or activities that contribute to the disease process downstream of UBQLN2 dysfunction could be inhibited with a variety of approaches. In one example, the protease of PEG10 could be inhibited with a small molecule [103], thereby preventing its self-cleavage and induced changes to gene expression. Together, these avenues provide a wealth of opportunity for therapeutic development and represent active areas of research.

## Conclusion and outlook

UBQLN2 is a widely expressed regulator of protein degradation via the UPS and autophagy that assists in regulating mitochondrial health and SG dynamics to maintain dendritic spines and neuronal health. Recent work highlighted here has detailed the effects of *UBQLN2* mutations on its many roles and how dysregulation of these pathways may contribute to the progression of ALS/FTD. Despite these advances, much remains unknown about the pathological role of UBQLN2 mutations in neurodegenerative diseases. Ultimately, continued research will enhance our understanding of neurodegenerative disease-associated *UBQLN2* mutations, allowing for the development of new therapeutics.

**Perspectives**

- UBQLN2 (Ubiquilin-2) is a shuttle factor which regulates protein degradation via the proteasome and autophagy, and is linked to neurodegenerative disease. In particular, mutation of UBQLN2 causes a familial form of amyotrophic lateral sclerosis (ALS)/ frontotemporal dementia (FTD). However, it remains unclear precisely how UBQLN2 mutation leads to neuronal dysfunction and symptoms of disease.
- Disease-linked UBQLN2 mutations likely act as both loss-of-function and a toxic gain-of-function to dysregulation of neuronal health. Critical changes include those to protein degradation, mitochondrial dynamics, stress granule regulation, and neuronal processes, thereby contributing to disease.
- Understanding how UBQLN2 mutation causes cellular dysfunction provides new avenues for therapeutic development, particularly in ALS/FTD. Particular focus is applied to areas of antisense oligonucleotide development, modulation of protein degradation pathways, and inhibition of dysregulated client activities.

## Abbreviations

ALS: amyotrophic lateral sclerosis
ASOs: antisense oligonucleotides
FTD: Frontotemporal dementia
SG: stress granule
STI1: stress-induced protein 1
Tg: transgenic
UBA: ubiquitin-associated domain
UBL: ubiquitin-like domain
UBQLNs: Ubiquilins
